# Editorial: World Health Day 2024: frontiers in public health presents: “My health, my right”

**DOI:** 10.3389/fpubh.2025.1679401

**Published:** 2025-08-29

**Authors:** Olatunde Aremu, Barbara Sawicka, Hubert Amu, María Del Carmen Valls Martínez, Tsitsi Masvawure

**Affiliations:** ^1^Department of Life and Sport Sciences, Birmingham City University, Birmingham, United Kingdom; ^2^Department of Plant Production Technology and Commodities Science, University of Life Sciences in Lublin, Lublin, Poland; ^3^Department of Population and Behavioural Sciences, Fred N. Binka School of Public Health, University of Health and Allied Sciences, Hohoe, Ghana; ^4^Mediterranean Research Center on Economics and Sustainable Development (CIMEDES), Economics and Business Department, University of Almería, Almería, Spain; ^5^Department of Integrative and Global Studies, Worcester Polytechnic Institute, Worcester, MA, United States

**Keywords:** World Health Day, wellbeing, my health, health for all, my right, health service utilization, WHO, universal coverage

World Health Day on April 7, 2024, calls for global commitment to the right of individuals to their health with the theme “My health, my right.” According to the World Health Organization (WHO), over half the world's population, more than 4.5 billion individuals, did not have access to essential health care, either in terms of physical or mental health, as of 2021 (1). This level of inequality introduces disparities which are shaped by gender, race, education, income, sexual orientation, place of residence, or physical disability (2, 3).

Frontiers in Public Health launched this Research Topic to commemorate the United Nations World Health Day 2024. This Research Topic, bringing together 13 articles, highlights the progress made, the impediments that persist, and the innovative approaches shaping the future of the right to health.

The World Health Organization (WHO) constitution, articulated for the first in 1946, the right to health. Specifically, it states that “the enjoyment of the highest possible standard of health is one of the fundamental rights of every human being irrespective of race, religion, political affiliation, economic status or social condition (4).” This statement by WHO was further enshrined as a human right in 1948 via the Universal Declaration of Human Rights and in 1966 in Article 12 of the International Covenant on Economic, Social and Cultural Rights (ICESCR), which encompasses four core areas—availability, accessibility, acceptability and quality (5). Health is indeed a fundamental human right, and it is important to recognize the need for legal responsibility, fairness, and non-discrimination in its production (5, 6). However, earlier efforts through universal health coverage (UHC), have provided little impetus toward improved healthcare access and impediments have remained in the realization of health for all (6). Although the theme “My health, my right” seems to lend support to an individual's sovereignty, by having a say and unhindered access to information that leads to decisions about their health, there are still challenges to the actualization of such. The advent of emerging and remerging infectious diseases of global health importance, lack of health literacy about availability of services, stigmatization of specific health problems, and cost of healthcare services adversely affect individuals' sovereignty to access health services and their right to health (7–10). Furthermore, the challenges arising from and posed by legislation around personal data privacy from the use of technological innovations have joined forces to make achieving the right to health for individuals somewhat difficult (8, 11).

The articles in this Research Topic address several themes that are relevant to advancing health as a human right. Some articles focus on wider socio-structural factors that impede the right to health, while others propose innovative approaches to ensuring that the right to health is attained for all. In the sections that follow, we offer a summary of the key messages from the articles in the Research Topic and show how they address the theme “My health, my right.”

## Social determinants of health and health inequalities

Several articles in this Research Topic focus on how social, economic, and environmental factors influence health outcomes and contribute to health inequalities. These articles focus specifically on the high prevalence of non-communicable diseases globally, which is negatively affecting individuals' rights to good health.

In the article by Zhang et al., the authors investigated the relationship between inequalities in chronic respiratory disease (CRD) and all-cause mortality in China and they found that individuals from poor backgrounds had substantially higher risks of chronic lung disease and asthma. This study highlights the need to address the root causes of economic inequalities, and the authors propose interventions that are aimed at improving the educational attainment of individuals from low socioeconomic backgrounds as the key solution.

The article by Liu et al., examines the global epidemiology of pulmonary arterial hypertension (PAH), focusing on trends in incidence, mortality, and disability-adjusted life years (DALYs) over a 32 year period to inform evidence-based policy and healthcare strategies. The findings from the study are novel and reveal an upward trajectory in the incidence of PAH, increasing from 23,301 in 1990 to 43,251 in 2021. Additionally, from 1990 to 2021, PAH-related deaths increased from 14,842 to 22,021. The authors recommend enhanced prevention and comprehensive management strategies as a strategy to shrink the global PAH burden and improve health equity.

The article by Cicekli and Eskin examined the prevalence and co-occurrence of lifestyle risk factors among university students and concluded that targeted interventions, such as promoting physical activity, providing affordable and nutritious meals, and educating students on healthy lifestyles, are essential to reducing non-communicable disease (NCD) risk among students. The study found that students at highest risk of developing NCDs lived in student housing, preferred eating fast foods and watched 4–5 h of television a day. Something about the socio-structural organization of university life seemed to increase students' risk of acquiring NCDs. The article by Cristiane Sibim et al. examined the potential interactions between socioeconomic indicators and the One Health Index (OHI) in South American countries and the authors found, surprisingly, that better environmental health was not associated with better human or animal health. The authors, as part of their findings, considered absence of stronger indicators for animal health to be a key gap in better understanding the interplay of One Health factors. A key finding the authors also highlight is that social factors, rather than economic factors like GDP, seem to explain differences in the One Health status observed in South American countries.

The last article we will highlight in this section is by Bezie et al. who investigated the prevalence of work-related burnout and its correlates among kindergarten teachers in Dessie City, Northeast Ethiopia. Results from bivariate logistic regression and multivariable regression models revealed the total prevalence of Work-related burnout (WRB) was 39.7% [95% CI (34.8, 44.6)]. Work–family conflicts, working conditions, perceived stress, job dissatisfaction, and kindergarten type were all significantly associated with WRB. Some of the social determinants of work-related burnout included long work hours and a lack of appreciation of the emotionally draining nature of kindergarten care. The authors recommend interventions focused on improving school environments to be more supportive of the wellbeing of teachers.

## Innovative approaches and interventions to address health disparities

Several articles in this Research Topic focus on the application of novel approaches, interventions, and best practices that can help combat health disparities, improve health equity, and safeguard the health rights of diverse people. The article by Sapp et al. discusses the development, implementation and evaluation of a pilot of two medical education interventions aimed at improving attitudes and empathy toward individuals with disability among medical students in the U.S. The findings show that one of the interventions, the elective course, but not the 2 hr course, significantly decreased student anxiety levels, likely due to more individual time working with individuals with disabilities. However, delayed analysis after 3 months showed that both interventions had a lasting impact on attitudes and behavior change when caring for individuals with disabilities. This study draws attention to the growing population of individuals with disabilities in the U.S. and the importance of ensuring that healthcare providers are well-trained to provide high quality services to this population.

The article by Chen and Tian assessed the financial effectiveness of a combination of modified gemcitabine and oxaliplatin in the management of gall bladder cancer (GBC) in China, using data from a randomized controlled study in individuals with metastatic GBC. The cost-effectiveness analysis concludes that in a Chinese healthcare context, modified gemcitabine coupled with oxaliplatin (mGEMOX) is not a cost-effective treatment option for unresectable GBC. This paper is important for highlighting the costs of medical treatments as key impediments to the realization of health for all.

Similarly, the article by Schenkman and Bousquat examined efficiency and effectiveness in health services in remote rural localities (RRL) compared to urban and rural communities of Brazil. The authors report that Brazilian RRL localities show superior resource and health efficiency largely due to how primary healthcare teams are organized. The authors concluded that reducing intersectional inequities in income and education by ethnicity could greatly increase the efficient attainment of health levels in society. This study is innovative in its attempt to quantify health provision and outcomes in the most remote of rural areas, which tend to be hard to reach and often not consistently included in national-level statistics. Remote rural communities often constitute some of the most invisible and vulnerable populations globally.

## Disparities in equity in access to healthcare services and outcomes among diverse populations

The importance of equity in access to, and utilization of healthcare services in improving the wellbeing of diverse populations cannot be overemphasized. In the article by Bu et al. the authors explored the heterogeneity of public health service use and how it relates to the social integration of older adult migrants in China. The authors concluded that many aspects influence utilization of public health services amongst older adult migrants, such as gender, education and extent of mobility. They also found that familiarity with local resources increased public health service utilization. Thus, the authors recommend more targeted policies that are user-friendly to help improve uptake of public health services for older adult migrants.

## Community empowerment and participation

The importance of initiatives that empower communities to advocate for their health rights, participate in decision-making processes, and contribute to improving health outcomes cannot be overstated. In the article by Borondy-Jenkins et al., the Hepatitis B Foundation (HBF) convened the first global hepatitis B and hepatitis delta Community Advisory Board (CAB) with 23 members from 17 countries, representing six out of the seven World Health Organization (WHO) regions, and countries with the largest hepatitis B and hepatitis delta disease burden. The aim was to reflect on the process of assembling an effective and motivated CAB and assess the impact on CAB participants. Three virtual focus group sessions were held with 16 participants in July and August 2023. Participants reported that through CAB membership, they gained networking and advocacy opportunities, as well as enhanced their knowledge of hepatitis B and hepatitis delta. The authors recommend that a regular internal review of the community advisory boards' structure and performance is critical to ensure the CAB is fulfilling its mission.

## Health policy and governance

Periodic assessment of the effectiveness of policies, strategies, and governance structures in promoting health equity and upholding the rights of individuals to health is essential. The article by Külper-Schiek et al. reported the outcome of an evaluation of the effectiveness of national immunization technical advisory groups (NITAGs) in middle-income countries funded by the WHO Regional Office for Europe and the Robert Koch Institute (RKI). The findings show that all the NITAGs studied lacked a well-staffed Secretariat to establish annual work plans and develop NITAG recommendations following a standardized process. The authors recommend that the WHO and NITAG partners continue to provide training on the standardized recommendation-making process and advocate for increased MoH support to NITAGs, including dedicated Secretariat staff. NITAGs play a critical role in ensuring easy access to life-saving immunizations and are thus key partners in the quest for health justice.

## Ethical dilemmas and considerations

Addressing ethical dilemmas that may arise in the provision of healthcare services is crucial to ensuring that individual rights are respected, and equitable health outcomes are maintained. The article by El Bouchikhi et al. is a scoping review that explores opportunities and ethical issues that are inherent in the use of digital technologies and the Internet of Things (IoT) within occupational safety and health (OSH). The review identifies many ethical issues but notes that these provide key information and guidance for those who wish to develop evaluation frameworks in line with a preventive regulatory approach. More importantly, the list informs policymakers and practitioners about the governance of such tools for ensuring more OSH.

## Conclusion: moving toward achieving the right to health

We, the Editors, strongly believe that the theme “My health, my right” is a call to renew action toward empowering individuals to achieve the needed right to health. The selection of articles from various regions globally present the current situation regarding the right to health from various dimensions.

Ultimately, to achieve the WHO declaration of equal rights to health and enhance individuals' long-term health and wellbeing, deep-rooted systemic injustice in access to healthcare, which manifests in several ways, must be fully eliminated (7, 12). Unparalleled and sustainable commitment to tackle disparities in access to and utilization of healthcare services for vulnerable groups must be pursued (3, 13). Health system strengthening is imperative and needed to ensure health equity; however, the need for multisectoral and community participation in spreading health awareness is essential and should be widely promoted (14). Participation requires enabling health service users, groups and civil society to engage in planning, decision-making, and implementation processes for health across all levels of the system (15). Literacy is vital in assimilation and comprehension of health information; hence, investment in such should be accorded a greater priority (16).

Advancements in the deployment of digital technologies have brought new hope in ensuring that people can access healthcare without geographical boundaries (17). Despite this, inequities persist along the lines of income, education, geography, and gender, disproportionately affecting vulnerable populations (2, 9, 10). The UHC, rooted in primary health care, can help countries accomplish the right to health by making sure all people have affordable, equitable access to health services (6). Notwithstanding all the efforts being made, it is ultimately the responsibility of individuals to take control of their health.

## References

[1] WHO. World Health Organization. World Health Day 2024: My Health, My Right (Geneva) (2024).

[2] Macnaughton G Ahmed AK. Economic inequality and the right to health: on neoliberalism, corporatization, and coloniality. Health Hum Rights. (2023) 25:105–10. 38145129 PMC10733770

[3] Germani F März JW Clarinval C Biller-Andorno N. Economic sanctions, healthcare and the right to health. BMJ Glob Health. (2022) 7:e009486. 10.1136/bmjgh-2022-009486 35896183 PMC9328087

[4] Binagwaho A Mathewos K. The right to health: looking beyond health facilities. Health Hum Rights. (2023) 25:133–5. 37266319 PMC9973503

[5] Amon JJ. Realizing the right to health: a long and winding road. Health Hum Rights. (2023) 25:1–14. 38145135 PMC10733759

[6] Harris J. UHC is the right goal, but is not the same as the right to health. Lancet Glob Health. (2023) 11:e1517. 10.1016/S2214-109X(23)00327-3 37734795

[7] Schrecker T. The COVID-19 pandemic as a tipping point: what future for the right to health? Health Hum Rights. (2023) 25:111–23. 38145142 PMC10733769

[8] Hill DC Andrade-Romo Z Solari K Adams E Forman L Grace D. COVID-19 vaccine equity and the right to health for displaced Venezuelans in Latin America. PLOS Glob Public Health. (2023) 3:e0001275. 10.1371/journal.pgph.0001275 36963074 PMC10021234

[9] GBD 2021 HAP Collaborators. Global, regional, and national burden of household air pollution, 1990-2021: a systematic analysis for the Global Burden of Disease Study 2021. Lancet. (2025) 405:1167–81. 10.1016/S0140-6736(24)02840-X 40118081 PMC11971481

[10] Espinosa MF Andriukaitis VP Kickbusch I Nishtar S Saiz E Takemi K. Realising the right to health for all people-UHC is the umbrella to deliver health for all. Lancet Glob Health. (2023) 11:e1160–1. 10.1016/S2214-109X(23)00202-4 37146627 PMC10154000

[11] Gruskin S. Health and human rights: what relevance now? Health Hum Rights. (2024) 26:7–10. 38933235 PMC11197873

[12] Chen Q Tian W Zheng L Li T. Safeguarding the right to health of the elderly in rural china: a legal analysis. Risk Manag Healthc Policy. (2023) 16:1621–32. 10.2147/RMHP.S420954 37621879 PMC10445640

[13] Moscoso A Piñones-Rivera C Arancibia R Quenaya B. The right to health care viewed from the indigenous research paradigm: violations of the rights of an Aymara. Health Hum Rights. (2023) 25:81–94. 37266320 PMC9973506

[14] Litalien É. Understanding the right to health in the context of collective rights to self-determination. Bioethics. (2021) 35:725–33. 10.1111/bioe.12938 34453349

[15] DiStefano MJ Karim SA Krubiner CB Hofman KJ. Integrating health technology assessment and the right to health in south africa: a qualitative content analysis of substantive values in landmark judicial decisions. J Law Med Ethics. (2023) 51:131–49. 10.1017/jme.2023.48 37226744

[16] General Comment and United Nations Human Rights Council. ‘The Right to Health' Fact Sheet No. 31 2008. New York, NY: United Nations Human Rights Council TRtHFSN, ed. (2008).

[17] Serrano Córdova C Torres I López-Cevallos D. Exploring the impact of Ecuador's policies on the right to health of Venezuelan migrants during the COVID-19 pandemic: a scoping review. Health Policy Plan. (2023) 38:1099–112. 10.1093/heapol/czad071 37572095 PMC10566316

